# Enhancement of Apple Stress Resistance via Proline Elevation by Sugar Substitutes

**DOI:** 10.3390/ijms25179548

**Published:** 2024-09-02

**Authors:** Zi-Quan Feng, Tong Li, Xin-Yi Li, Long-Xin Luo, Zhi Li, Chun-Ling Liu, Shun-Feng Ge, Zhan-Ling Zhu, Yuan-Yuan Li, Han Jiang, Yuan-Mao Jiang

**Affiliations:** National Research Center for Apple Engineering and Technology, Shandong Collaborative Innovation Center of Fruit & Vegetable Quality and Efficient Production, College of Horticulture Science and Engineering, Shandong Agricultural University, Tai’an 271018, China; 2019110237@sdau.edu.cn (Z.-Q.F.); 17861507291@163.com (T.L.); 17860295482@126.com (X.-Y.L.); fuluobei0601@163.com (L.-X.L.); 17660947118@163.com (Z.L.); chunlingliu23@126.com (C.-L.L.); geshunfeng210@126.com (S.-F.G.); zhlzh@sdau.edu.cn (Z.-L.Z.); liyuanyuan@sdau.edu.cn (Y.-Y.L.)

**Keywords:** sugar substitute, antiretroviral activator, proline

## Abstract

Plants encounter numerous adversities during growth, necessitating the identification of common stress activators to bolster their resistance. However, the current understanding of these activators’ mechanisms remains limited. This study identified three anti-stress activators applicable to apple trees, all of which elevate plant proline content to enhance resistance against various adversities. The results showed that the application of these sugar substitutes increased apple proline content by two to three times compared to the untreated group. Even at a lower concentration, these activators triggered plant stress resistance without compromising apple fruit quality. Therefore, these three sugar substitutes can be exogenously sprayed on apple trees to augment proline content and fortify stress resistance. Given their effectiveness and low production cost, these activators possess significant application value. Since they have been widely used in the food industry, they hold potential for broader application in plants, fostering apple industry development.

## 1. Introduction

Adverse environments, such as drought, salinity, cold, and high temperatures, are the primary factors affecting plant growth and development [1]. When these extreme conditions surpass the maximum tolerance threshold of plants, they not only disrupt normal physiological processes like respiration and photosynthesis but can also lead to plant death in severe cases.

Salinity stress is one of the most prevalent abiotic stresses in agricultural production, particularly for plants cultivated in saline soils. Changes in external Na^+^ concentrations affect numerous aspects of plant physiology and metabolism. High Na^+^ concentrations can disrupt water and ion homeostasis in plant cells, resulting in plant depression [2]. Na^+^ in salt solutions prevents water uptake by lowering the free energy of water and reduces nutrient production and conversion rates, thereby stunting plant growth.

Although the impact of salt stress on plant growth is significant, it is primarily a concern in specific saline soil environments and does not pose the most significant threat to agricultural production. In nature, plants are more susceptible to natural disasters like drought, which can threaten any plant on land at any time [3], causing extensive damage to global agricultural production. When plants are subjected to environmental stressors such as early drought, reactive oxygen radicals (RORs) like H_2_O_2_ and O_2_^−^ rapidly accumulate in plant leaves, exerting a potent toxic effect on cells [4]. These RORs can cause membrane lipid peroxidation or degreasing, forming malondialdehyde (MDA), which reduces membrane stability and disrupts the structure and function of various biofunctional molecules within the cell [5]. Drought stress hinders plant growth; a drastic drop in soil moisture increases soil hardness and significantly inhibits the development of the root system, resulting in a plant water imbalance [6].

Low temperature is a significant environmental factor that restricts the growth and development of fruit trees [7]. There are three fundamental temperature requirements for plant growth: minimum, maximum, and optimum temperatures. When the ambient temperature drops below the minimum temperature, the plant experiences low temperature stress [8]. This stress can have numerous detrimental effects on plants, such as seed germination inhibition, stunted growth, reduced reproduction, and reduced crop yield and quality [9,10,11]. Cold temperatures can alter the fluidity of cell membranes, thereby affecting the function of membrane-localized proteins and triggering subsequent responses. Prolonged exposure to severe cold can also impact the selective permeability of cell membranes, leading to membrane dysfunction [12]. Freezing conditions cause water to form ice, which dehydrates cells, and extreme cold temperatures can significantly affect enzyme activity, including those involved in ROS scavenging, causing oxidative stress. This, in turn, disrupts photosystem activity and induces stomatal closure, thereby reducing photosynthesis in plants [13].

Proline (Pro), a common component of plant proteins, is abundant in the free state throughout the plant body. Under adverse conditions such as drought, salinity, heat, cold, and freezing, proline content in plants significantly increases [14,15]. Proline levels in plants partially indicate the plant’s resistance to stress. Proline acts as an osmotic regulator in plant cytoplasm, contributing to the stabilization of biomolecule structure, reducing cellular acidity, alleviating ammonia toxicity, and regulating cellular redox as an energy reservoir [16]. Additionally, proline’s extreme hydrophilicity stabilizes protoplasmic colloids and metabolic processes in tissues, lowering the freezing point and preventing cell dehydration [17]. This increase in proline under low-temperature conditions improves the cold hardiness of plants. In living organisms, proline is not only an optimal osmoregulator but also functions as a membrane and enzyme protector and free radical scavenger, thereby safeguarding plant growth under stress [18].

The apple, a globally significant fruit tree, is renowned for its ecological adaptability, nutritional value, ease of storage, and long shelf life. It is the predominant fruit consumed worldwide, particularly in countries such as China, which is the world’s largest producer and consumer, accounting for over half of global production and acreage. However, the increasing severity and frequency of extreme weather events, exacerbated by climate change, pose challenges to apple production. With the continued advancement of the growing season and the flowering period of apples, the risk of cold and frost damage to fruit trees due to low temperatures has also increased.

Moreover, the year-on-year expansion of saline soils in China, particularly in coastal, arid, and semi-arid regions like Jiangsu, Shandong, and Hebei, is a significant concern. These areas experienced low rainfall, uneven seasonal distribution, sparse vegetation cover, and soil erosion, which consequently threaten the sustainable development of the apple industry in the region. Therefore, improving apple plant resistance to these adverse environmental conditions and enhancing yield and fruit quality under the influence of salinity and low temperature stress has become a pressing issue in apple-producing areas.

Sugar substitutes, known as sweeteners, are substances that offer sweetness with minimal to no caloric content [19]. They are typically classified into two main groups: synthetic and natural. Synthetic sugar substitutes, including aspartame, sucralose, and saccharin, are chemically produced compounds that impart sweetness. Aspartame is a common ingredient in sugar-free beverages and low-calorie foods [20]. Natural sugar substitutes include plant-derived ingredients like erythritol and rosmarinic acid sweeteners, which have a sweet taste. Sugar substitutes are significantly sweeter than sucrose (table sugar) and only a small amount is needed to achieve the same level of sweetness. The low or non-existent caloric value of sugar substitutes aids in reducing overall calorie intake, benefiting weight management and preventing obesity. For instance, low-sugar pastries made with sugar substitutes instead of sucrose allow individuals focusing on weight management to enjoy occasional sweet treats without consuming excessive calories. Additionally, sugar substitutes do not cause significant blood glucose fluctuations and help maintain stable blood glucose control for those with diabetes or high blood glucose levels [21]. Given the widespread use of sugar substitutes in food and beverages, we aimed to explore whether they could play a similar role in plants.

In the absence of existing research on the impact of sugar substitutes on plants, we are exploring whether these substitutes influence the growth and development of apple seedlings. We have selected three different sugar substitutes. Our findings reveal that these substitutes, when applied at low concentrations, further increased the plant proline content. Moreover, exposure to adversity increased their resistance to various stresses. This enables the seedlings to sustain optimal growth even under adversity.

## 2. Results

### 2.1. Low Concentration of Sugar Substitutes Promote Proline Accumulation

**Figure 1:**
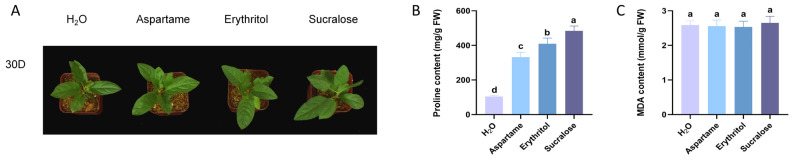
Phenotypes after 30 days of sugar substitute treatment. (**A**) Apple growth status after 30 days of sugar substitute treatment. (**B**) Proline content after 30 days of sugar substitute treatment. (**C**) MDA content after 30 days of sugar substitute treatment. Error bars represent the standard deviations (n = 3). Different letters above the bars indicate significantly different values (*p* < 0.05).

We subjected apple seedlings to lower concentrations of aspartame, sucralose, and erythritol for 30 days, with a control group receiving only water. No significant differences in plant growth were observed after this period (Figure 1A). Interestingly, further measurements of important plant stress tolerance indicators showed a significant increase in proline content in the treated seedlings (Figure 1B), with the trichlorosucrose group increasing the most. Conversely, malondialdehyde content remained unchanged in all groups (Figure 1C).

### 2.2. Low Concentration of Sugar Substitutes Improves Salt Resistance

**Figure 2:**
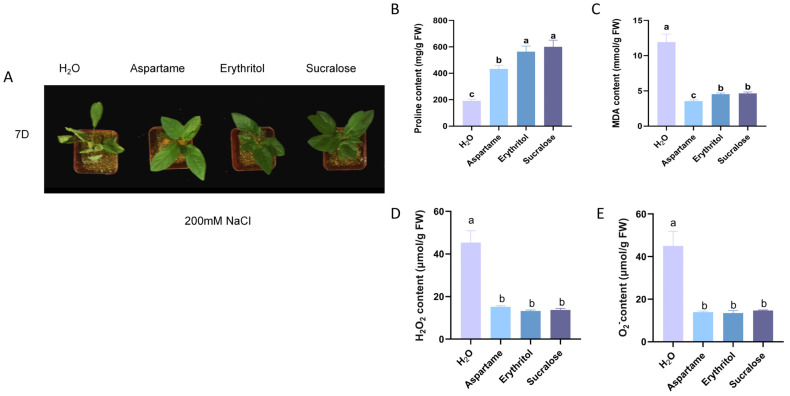
200 mM NaCl treatment 7 days after apple phenotype. (**A**) Apple growth status after 7 days of 200 mM NaCl treatment (**B**) Proline content after 7 days of NaCl treatment. (**C**) MDA content after 7 days of NaCl treatment. (**D**) H_2_O_2_ content after 7 days of NaCl treatment. (E) O_2_^−^ content after 7 days of NaCl treatment. Error bars represent the standard deviations (n = 3). Different letters above the bars indicate significantly different values (*p* < 0.05).

Studies have shown the significant role of proline in enhancing plant resistance to osmotic stress [22]. Given that the above reagents can augment proline content without impeding growth, we initially exposed the plants to a lower concentration for ten days, subsequently subjecting them to 200 mM NaCl for seven days. The control group using clear water exhibited severely impaired growth, whereas all treatment groups demonstrated enhanced resistance to salt stress (Figure 2A). Furthermore, physiological measurements revealed that aspartame, erythritol, and sucralose all elevated proline content and reduced malondialdehyde content, with erythritol and sucralose showing stronger effects than aspartame (Figure 2B,C). Reactive oxygen species measurements in the plants revealed that all three substances significantly decreased the levels of hydrogen peroxide and superoxide anion, with no significant differences observed among the three (Figure 2D,E).

### 2.3. Low Concentration of Sugar Substitutes Enhances Cold Tolerance in Plants

**Figure 3:**
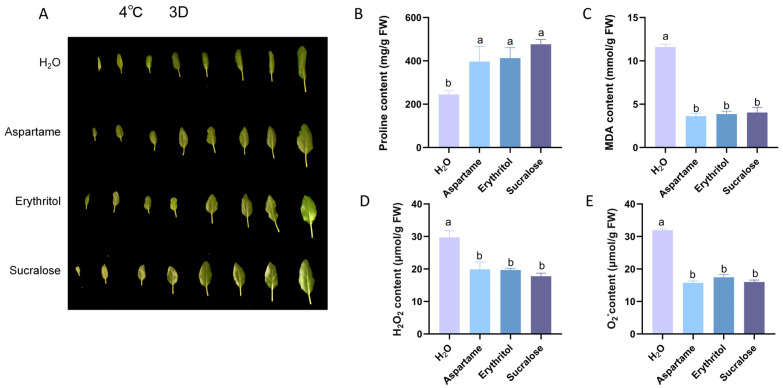
4 °C treatment three days after apple phenotype. (**A**) Apple growth status after 3 days of 4 °C treatment (**B**) Proline content after 3 days of 4 °C treatment. (**C**) MDA content after 3 days of 4 °C treatment. (**D**) H_2_O_2_ content after 3 days of 4 °C treatment. (**E**) O_2_^−^ content after 3 days of 4 °C treatment. Error bars represent the standard deviations (n = 3). Different letters above the bars indicate significantly different values (*p* < 0.05).

In the northern regions of China, cold attacks in spring often cause damage to young apple leaves. We subjected them to cold stress treatment to simulate the damage of inverted spring cold. The results showed that the leaves showed strong cold resistance after applying a sugar substitute (Figure 3A). Physiological index measurements revealed that proline content increased, while malondialdehyde and reactive oxygen species content decreased, with these effects being closely related (Figure 3B–E). Meanwhile, gene expression analysis of cold stress-related genes *MdCBF1/2/3* and *MdKIN1* in apple seedlings showed up-regulation (Figure S2), indicating that the sugar substitute improved cold tolerance through the CBF pathway.

### 2.4. Low Concentration of Sugar Substitutes Enhances Drought Tolerance in Plants

**Figure 4:**
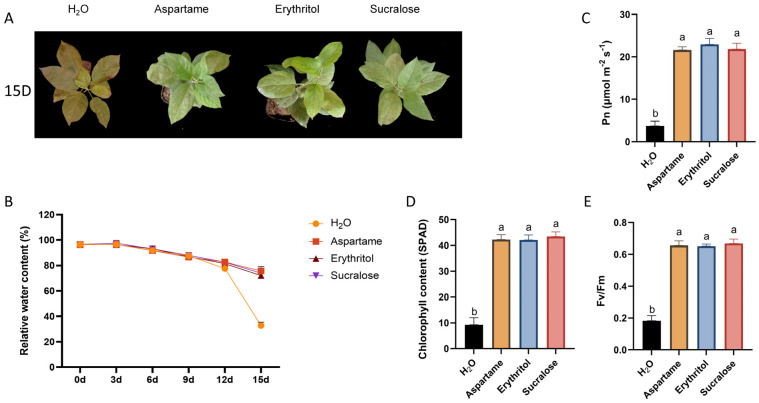
Drought treatment 15 days after apple phenotype. (**A**) Apple growth status after 15 days of drought treatment (**B**) Relative water content after 15 days of drought treatment. (**C**) Pn after 15 days of drought treatment. (**D**) SPAD after 15 days of drought treatment. (**E**) Fv/Fm after 15 days of drought treatment. Error bars represent the standard deviations (n = 3). Different letters above the bars indicate significantly different values (*p* < 0.05).

Drought is a significant yield and quality limiter for crops, and improving drought tolerance can be achieved through breeding and cultivation techniques, with cultivation being easier and quicker [23]. Given the previous finding that the sugar substitute increased proline content (Figure 1B), we hypothesized that it could also enhance water content, thereby improving drought resistance. We watered seedlings with the treatment solution every 3 days and subjected them to a contact drought treatment for 15 days. The results showed that the treated apple seedlings exhibited significantly improved drought tolerance (Figure 4A) and higher leaf water content compared to the untreated control (Figure 4B). Further measurements of leaf photosynthetic capacity revealed that the chlorophyll content (spad), Pn, and FV/FM of treated leaves were higher than those of the control (Figure 4C–E), indicating that the sugar substitute treatment could maintain the photosynthetic capacity of apple leaves under drought stress.

### 2.5. Low Concentration of Sugar Substitutes Enhances Plant Disease Resistance

**Figure 5:**
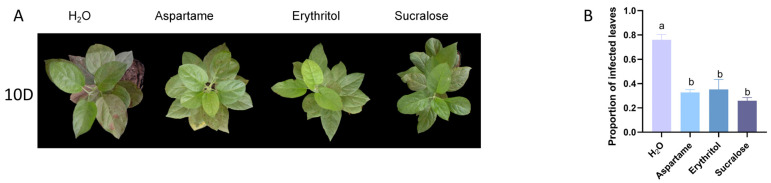
Apple phenotype after 10 days of growth in a diseased environment. (**A**) Apple growth status after 10 days in a diseased environment. (**B**) Proportion of susceptible leaves after 10 days in a diseased environment. Error bars represent the standard deviations (n = 3). Different letters above the bars indicate significantly different values (*p* < 0.05).

Disease poses a significant challenge for apple growers, particularly in greenhouse environments where apple seedlings are cultivated [24]. Preliminary tests revealed that plants treated with the treatment solution had less disease and better growth. To further investigate, we re-treated seedlings using a water-sprayed control group and incubated them in a rust environment for 10 days. The treatment group sprayed with sugar substitute exhibited significantly less leaf susceptibility to the disease compared to the untreated group (Figure 5A). Statistical results showed that its leaf incidence was approximately one-third the leaf incidence of the untreated group (Figure 5B).

### 2.6. The Effect of Sugar Substitutes on the Quality of Apple Fruits

**Figure 6:**
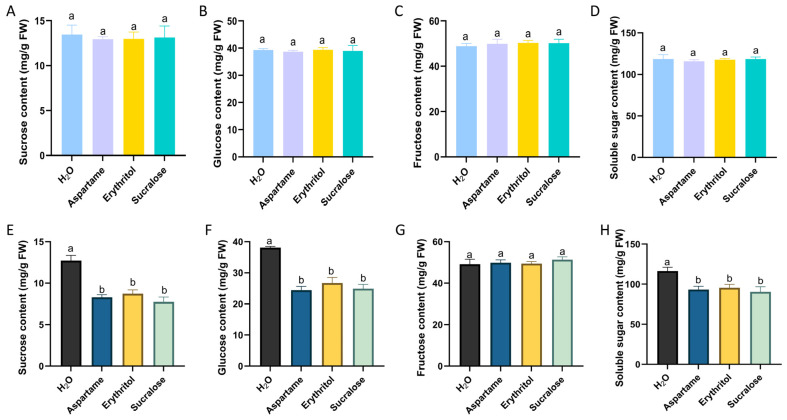
Fruit sugar content after sugar substitute treatment. (**A**) Sucrose content of fruits after treatment with low concentrations of sugar substitutes. (**B**) Glucose content of fruits after treatment with low concentrations of sugar substitutes. (**C**) Fructose content of fruits after treatment with low concentrations of sugar substitutes. (**D**) Soluble sugar content of fruits after treatment with low concentrations of sugar substitutes. (**E**) Sucrose content of fruits after treatment with high concentrations of sugar substitutes. (**F**) Glucose content of fruits after treatment with high concentrations of sugar substitutes. (**G**) Fructose content of fruits after treatment with high concentrations of sugar substitutes. (**H**) Soluble sugar content of fruits after treatment with high concentrations of sugar substitutes.

Sugar substitutes have been shown to influence sugar metabolism in animals, despite not being directly involved in metabolic processes [25,26,27]. We sought to determine whether the application of sugar substitutes would affect sugar metabolism processes in fruits and, consequently, fruit quality. Using two spraying concentrations, we found no differences in main sugars between the treatments and the clear water control in the fruit (Figure 6A–D). However, the group using the higher concentration showed a decrease in sucrose and glucose content, leading to a reduction in total soluble sugars (Figure 6E,F) but no effect on fructose content (Figure 6G). These findings suggest that the lower concentration of sugar substitute not only enhances apple tree resistance to adversity but also has no adverse effect on fruit quality.

## 3. Discussions

Salt stress significantly damages plants, but numerous studies have shown that plants have evolved protective mechanisms to mitigate this damage. One such consequence of salt stress is membrane lipid peroxidation, which leads to the MDA accumulation, a reliable indicator of stress tolerance as it reflects membrane integrity [28]. In our study, plants treated with a sugar substitute exhibited better growth under salt stress (Figure 2A), showing higher Pro content and lower MDA levels compared to untreated plants (Figure 2B,C). This suggests that the sugar substitute helps increase Pro content, thereby maintaining intracellular osmotic balance and preventing membrane damage. Environmental stresses, such as salt, often induce the production and accumulation of excessive ROS, which cause oxidative damage to cellular components [29]. To clarify the physiological mechanism behind the enhanced salt tolerance, we compared ROS levels between treated and control groups. The sugar substitute treatment effectively scavenged excessive ROS (Figure 2D,E), further contributing to increased salt stress tolerance.

The peroxidation of cell membrane lipids is closely linked to cold tolerance in plants [30]. The plant cell membrane system is the primary target of low-temperature injury under low-temperature stress. In early spring, northern China often experiences cold spells that severely impact agricultural production. This study demonstrates that preemptive application of sugar substitutes before the onset of low temperatures can mitigate cold-induced damage (Figure 3A). Further studies confirmed that spraying sugar substitute increased Pro content while reducing MDA and ROS levels. These findings align with previous research on salt stress, indicating that sugar substitutes enhance plant resistance under various stress conditions.

Drought stress is a significant obstacle to sustainable agriculture, impacting numerous physiological and biochemical processes in plants. Specifically, drought inhibits plant growth by reducing photosynthesis, leading to metabolic disorders, cellular structural disruption, and impaired enzyme activity and hormonal homeostasis [31]. In our study, we demonstrated that drought tolerance in plants can be significantly enhanced by applying sugar substitutes (Figure 4A). These substitutes significantly inhibit chlorophyll degradation under drought conditions, thereby maintaining high photosynthetic capacity (Figure 4D). Additionally, drought conditions exacerbate disease in apples. Notably, we observed that spraying pronioside improves plant disease resistance and reduces the proportion of disease-susceptible leaves (Figure 5A), suggesting a potential mechanism by which pronioside enhances disease resistance, warranting further investigation.

Previous animal studies have indicated that sugar substitutes, while not directly involved in metabolism, can influence glucose metabolism [25,26,27]. To explore whether a similar effect occurs in plants, we initially sprayed fruits with the same concentration of sugar substitutes used on leaves. This treatment did not alter sugar levels in the fruit (Figure 6A–D). However, when we applied a higher concentration, we observed a reduction in glucose and sucrose levels, leading to a decrease in the total soluble sugar content in the fruits (Figure 6E,F). Furthermore, spraying apples with higher concentration of pronioside inhibited plant growth (Figure S3). We hypothesize that pronioside exhibits a concentration-dependent effect, where lower concentrations improved plant resistance to various stresses without affecting fruit sugar content, while higher concentrations interfere with sugar metabolism and inhibit plant growth.

Water is crucial for plant growth and development, yet various environmental stresses can induce cellular osmotic stress and destabilize cell membranes [32]. In this study, we observed that spraying pronioside during the plant growth phase promotes proline accumulation, helping maintain osmotic pressure balance across cell membranes (Figure 1B). Additionally, pronioside demonstrated a role in enhancing disease resistance (Figure 5A). Further studies showed that this applied pronioside concentration did not interfere with sugar metabolism in the fruit. These results indicate that the three sugar substitutes can activate plant resistance to stress at lower concentrations without compromising fruit quality (Figure 6A–D), indicating their potential as valuable plant stress activators. We also found that applying low concentrations of sugar substitutes to apples increases their proline content without inhibiting apple growth. When plants are subjected to various stresses, such treatments improve their resistance to stress by increasing the proline content, while not adversely affecting apple quality. Although we established that sugar substitution treatment increases proline content in apples, the precise biological mechanism remains unclear and will be the focus of future research.

## 4. Materials and Methods

### 4.1. Plant Materials and Growth Conditions

The test material consisted of 60-day-old apple (*Malus domestica*) M9T337 dwarf autogenous rootstock seedlings, each ~15 cm tall and exhibiting uniform growth [33]. The seedlings were cultivated under natural light conditions: 28/18 °C (daytime) and 15/10 °C (nighttime) [34].

### 4.2. Fresh Weight

Before the experiment, the plants were rinsed once with tap water, followed by deionized water, and then dried. The fresh weight of each plant part was measured using an electronic balance.

### 4.3. Proline Content

For proline extraction, an appropriate amount of plant material was weighed, chopped, and placed in a mortar. A 3% sulfosalicylic acid solution was added, and the mixture was ground into a homogenate. This homogenate was then transferred into a centrifuge tube and centrifuged at 4000 rpm for 10 min, yielding the supernatant as the proline extract. Next, 2 mL of the supernatant was pipetted into a graduated tube with a stopper, to which 2 mL of glacial acetic acid and 2 mL of acidic ninhydrin reagent were added. The mixture was shaken thoroughly and heated in a boiling water bath for 30 min. After cooling, 4 mL of toluene was added, and the mixture was shaken again to extract the red substance. Once the solution had separated into layers, the toluene layer was transferred to a cuvette for absorbance measurement at 520 nm using a spectrophotometer (Beijing Solarbio Science & Technology Co., Ltd., Beijing, China, BC0290) [35].

### 4.4. Malondialdehyde Content

Malondialdehyde (MDA) reacts with thiobarbituric acid (TBA) to form a red product with a maximum absorption at 532 nm. The MDA content can be determined by measuring the absorbance difference between 532 nm and 600 nm [36].

### 4.5. Hydrogen Peroxide Content

For the detailed procedure, a previous method was referred to [33]. Hydrogen peroxide (H_2_O_2_) reacts with titanium sulfate, producing a yellow titanium peroxide complex with a characteristic absorption at 415 nm.

### 4.6. Superoxide Anion Radical Content

Similarly, superoxide anion reacts with hydroxylamine hydrochloride to form nitrite (NO_2_^−^), which then reacts with α-naphthylamine to produce a red azo compound, absorbing maximally at 530 nm.

### 4.7. Extraction of Plant Genomic RNA

RNA samples were collected three days post-treatment and preserved in RNA preservation solution (RNAfollow M6100, New Cell & Molecular Biotech, Newcastle upon Tyne, UK) at ultra-low temperatures before gene expression analysis. Plant RNA was extracted using the Omni Plant RNA Kit with tDNase I (RC411, Vazyme, Nanjing, China). The synthesized cDNA served as a template for quantitative real-time PCR (qRT-PCR) to assess the expression levels of selected genes, using apple 18S rRNA as a control. Primers were designed using Primer3Plus (https://primer3plus.com/cgi-bin/dev/primer3plus.cgi, accessed date on 1 May 2024), and all sequences are listed in Supplemental Table S1. Each qRT-PCR measurement was performed in triplicate, and relative gene expression was calculated using the 2^−ΔΔCt^ method [37].

### 4.8. Photosynthetic Rate and Chlorophyll Fluorescence

Net photosynthetic rate (Pn) was measured using a portable photosynthetic assay system (CIRAS-3, PPSystems, Amesbury, MA, USA) on a sunny day between 9:00 and 11:00 p.m. Functional leaf blades at identical positions were selected for the measurements. The maximum photochemical efficiency (Fv/Fm) was assessed using a closed chlorophyll fluorescence imaging system (FluorCam, PSI, Brno, Czech Republic).

### 4.9. Relative Leaf Water Content

Leaf samples were cleaned and weighed to determine their fresh weight (W1). Subsequently, the leaves were soaked in water for 12 h to achieve saturation, after which the saturated weight (W2) was measured. The leaves were then dried, and the dry weight (W3) was recorded. The relative water content (RWC) of the leaves was calculated using the formula: (W1 − W3)/(W2 − W3).

### 4.10. Sugar Content

The soluble sugar content, including sucrose, fructose, and glucose, in red star apple fruits was determined as follows [38]: (1) First, 1–2 g of plant material was weighed using a mortar and pestle. (2) Then, 2 mL of 80% ethanol was added to the material, ground thoroughly, and transferred to a 50 mL centrifuge tube. The volume was adjusted to 15 mL with 80% ethanol. (3) The mixture was extracted at 75 °C for 30 min in a water bath. (4) It was centrifuged at 2800× *g* for 10 min. (5) The supernatant was transferred to a fresh 50 mL centrifuge tube. (6) The supernatant was dried in an oven at 60 °C for 2–3 days. The residue was reconstituted with ddH_2_O, mixed thoroughly, and the volume was adjusted to 10 mL. (7) The extract was filtered through a 0.22 μm microfilter, and the soluble sugar content was determined using a Beckman P/ACE capillary electrophoresis system (Beckman Instruments Inc., Palo Alto, CA, USA) [39].

### 4.11. Data Analysis

Each experiment was independently performed in triplicate unless stated otherwise. The data are expressed as mean ± standard deviation. Statistical analysis was conducted using one-way ANOVA, followed by Duncan’s multiple range test for mean comparisons. Significant differences were indicated by different letters at the *p* < 0.05 level.

## 5. Conclusions

Aspartame, erythritol, and sucralose can increase proline levels in plants at low concentrations, enhancing their resistance to various stresses under adverse conditions. While each of these sugar substitutes have different resistance effects on different stresses, they all share the ability to activate plant resistance at low concentrations and inhibit growth at higher concentrations. When applied at low concentrations to fruit, these sugar substitutes do not compromise fruit quality. Therefore, low-concentration sugar substitutes can be used as pre-treatments to bolster plant resistance before exposure to stress.

## Data Availability

The data supporting the findings of this manuscript are available from the corresponding authors upon reasonable request.

## Supplementary Materials

The following supporting information can be downloaded at: https://www.mdpi.com/article/10.3390/ijms25179548/s1.
