# Supplementary material for: Enhancement of Apple Stress Resistance via Proline Elevation by Sugar Substitutes
Source: Int J Mol Sci. 2024 Sep 2;25(17):9548. doi: 10.3390/ijms25179548 (PMC11395137; doi:10.3390/ijms25179548)
Supplement: Supplementary file 1 [file ijms-25-09548-s001.zip › ijms-3142848-supplementary.pdf]

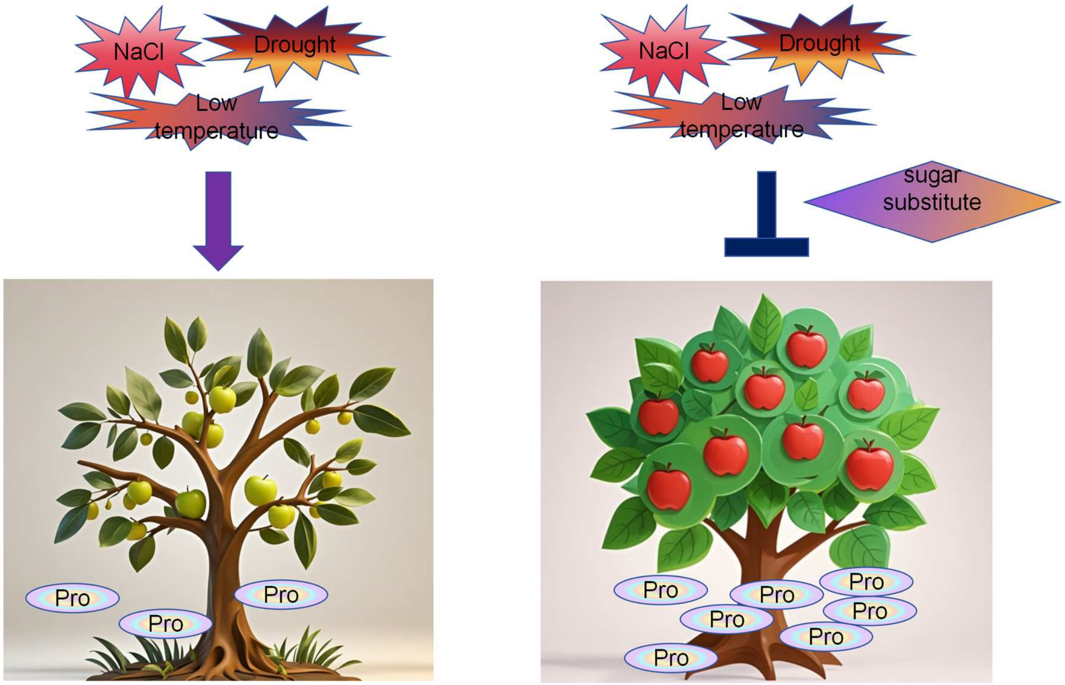

Pattern diagram of sugar substitutes improving Apple Resistance

Table S1. qRT-PCR primers used in this study.

Primer name	Primer sequence_F (5’-3’)	Primer sequence_R (5’-3’)
Md18s	ACACGGGGAGGTAGTGACAA	CCTCCAATGGATCCTCGTTA
MdCBF1	GAGGAGTCGGATGAGGTTTGT	CCACGTCATCATCATCACTGTA
MdCBF2	GGAGGCTGCAGAGACGTTC	CAGCAACCTTGGCATGTCG
MdCBF3	GAGGTCATTTTGGCGTCCA	TGGTTCTCTCATTTCGCACAC
MdKIN1	ATTTCGGATTATGGATACCAAGTTG	TAACGAACACGTACAGATTGCCTTT
MdSOS1	GTTGGCATGTTGGAAGAA	CCGTTAGAGGATGGAGAC
MdSOS2	CAAAAGCACCATTCTCAAGCAC	CCGACCAGCCAAAACCTCT
MdSOS3	AAGGCAAGGCGGCAGTTT	GCGAGGCATTGGGATGAA
MdDREB2A	AATTCAGGATCGGAGCAAGTAT	AGCTGGTACGACAAATTTGATG

MdNAC143	GGTACCATGAGCGGAGGAGGAGACGAG	GGATCCAAAATTGGGTGTGGAGATAGGA
MdbHLH122	CACCAATTGGCTCTTCGATT	ACACAACCCAATCTGGCACT
MdbHLH130	TCTGGCTAGTGAAGGTGGTG	GACGACGGCTGGTAAAATCC

Figure legends

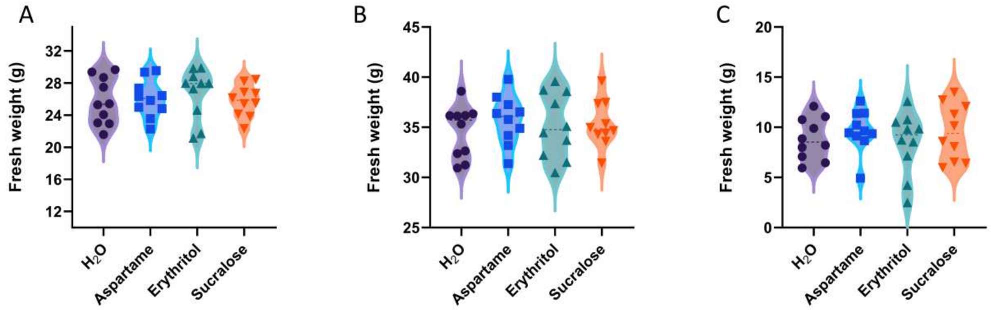

Figure S1. Change in fresh weight before and after 30 days of sugar substitute treatment

(A) Fresh weight of apples before sugar substitute treatment.(B) Fresh weight of apples after 30 days of sugar substitute treatment. (C) Increase in apple fresh weight in 30 days of sugar substitute treatment. n=10.

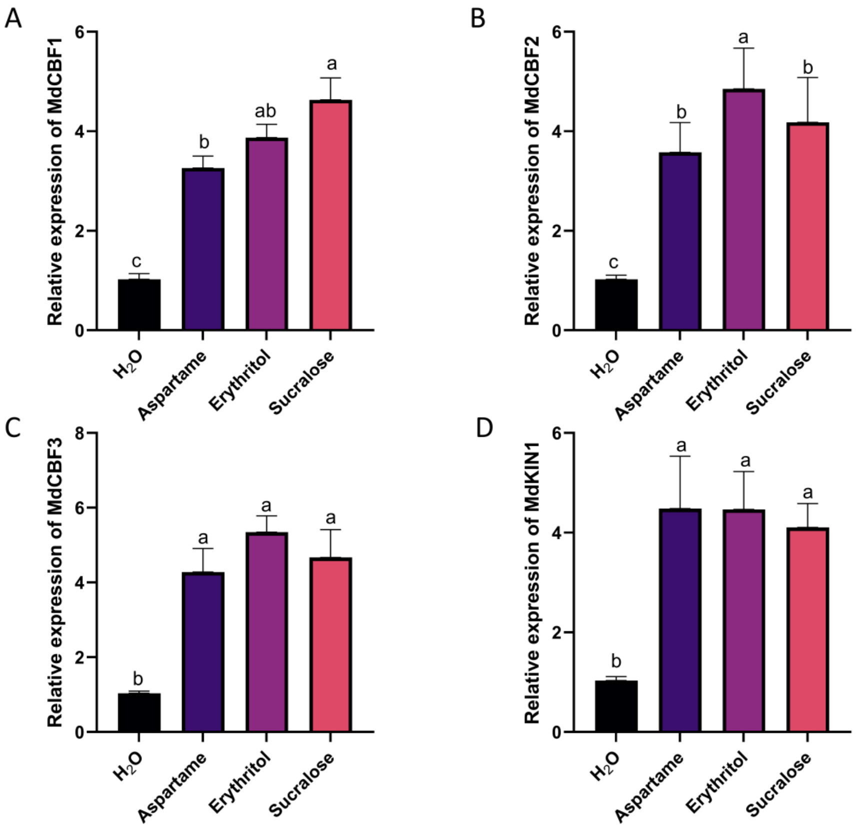

Figure S2. Expression of relevant genes after cold treatment

(A) MdCBF1 gene expression between different surrogate sugar treatments. (B) MdCBF2 gene expression between different surrogate sugar treatments. (C) MdCBF3 gene expression between different surrogate sugar treatments. (D) MdKIN1 gene expression between different surrogate sugar treatments. Error bars represent the standard deviation (n=3). Different letters above the bars indicate significantly different values ($P < 0.05$).

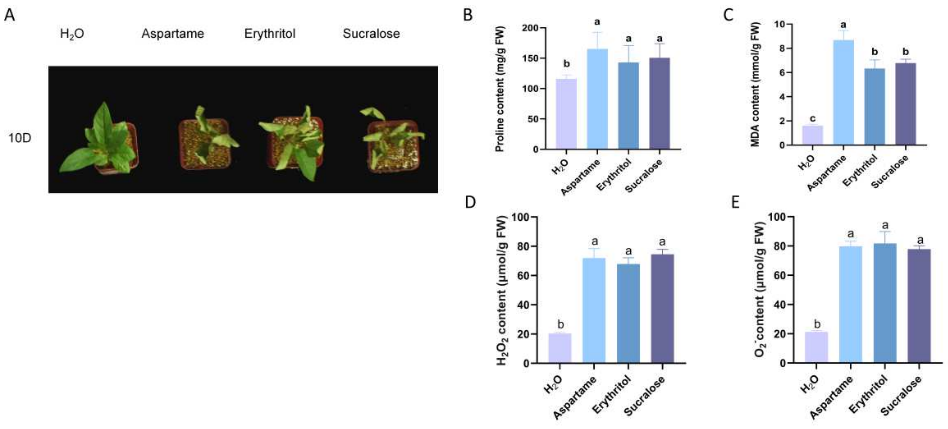

Figure S3. Growth status of apple seedlings after high concentration of sugar substitute treatment

(A) Apple growth status after 10 days of high concentration of sugar substitute treatment. (B) Proline content after 10 days of high concentration of sugar substitute treatment. (C) MDA content after 10 days of high concentration of sugar substitute treatment. (D) H_2O_2 content after 10 days of NaCl treatment.(E) O_2^- content after 10 days of high concentration of sugar substitute treatment. Error bars represent the standard deviation (n=3). Different letters above the bars indicate significantly different values ($P < 0.05$).

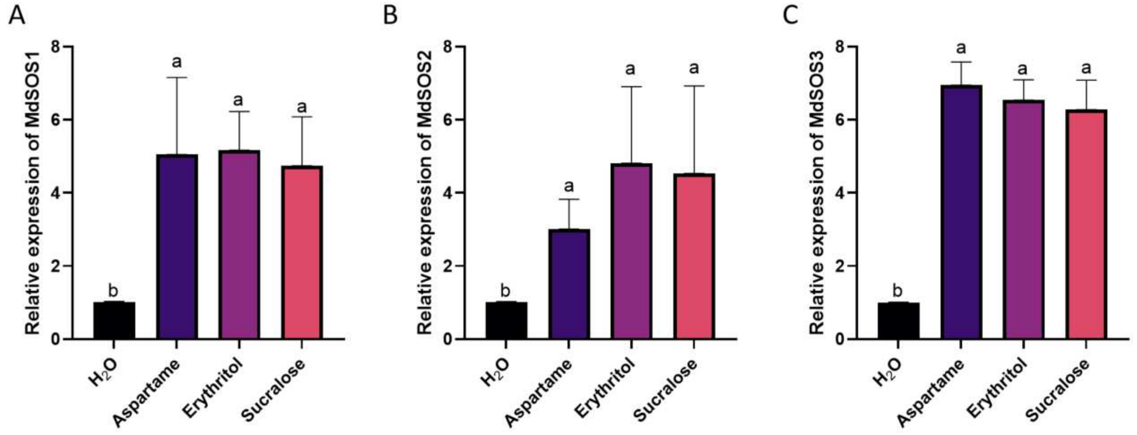

Figure S4. Expression of relevant genes after salt treatment

(A) MdSOS1 gene expression between different surrogate sugar treatments. (B) MdSOS2 gene expression between different surrogate sugar treatments. (C) MdSOS3 gene expression between different surrogate sugar treatments. Error bars represent the standard deviation (n=3). Different letters above the bars indicate significantly different values ($P < 0.05$).

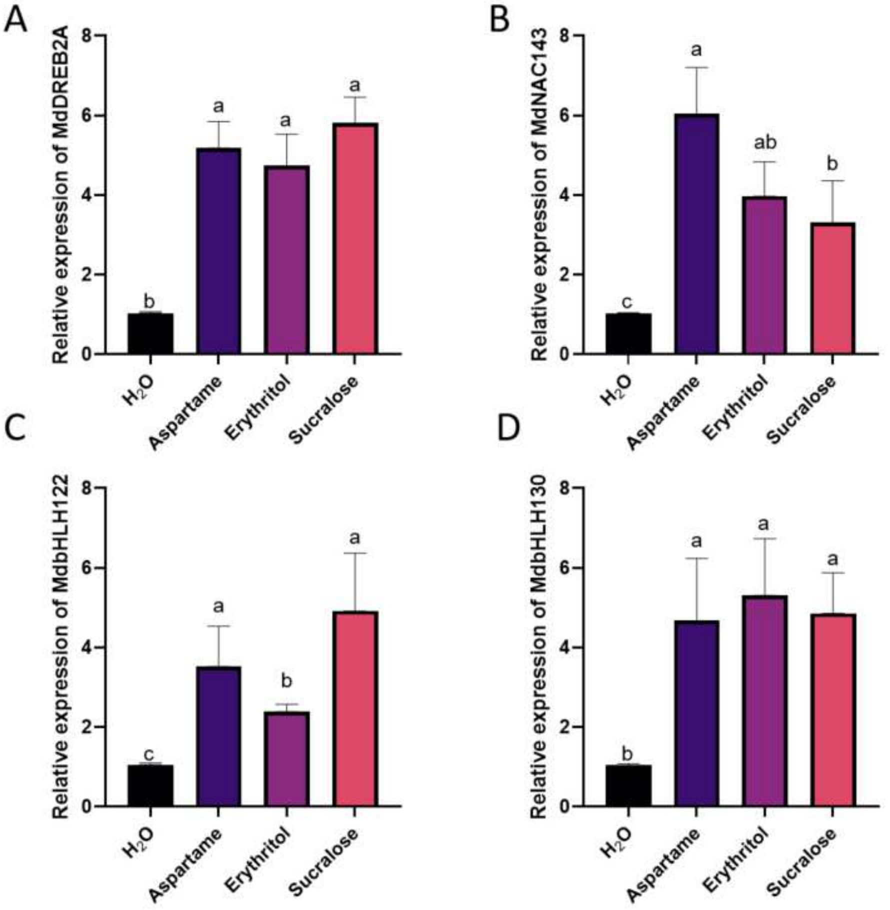

Figure S5. Expression of relevant genes after drought treatment

(A) MdDREB2A gene expression between different surrogate sugar treatments. (B) MdNAC142 gene expression between different surrogate sugar treatments. (C) MdbHLH122 gene expression between different surrogate sugar treatments. (D) MdbHLH130 gene expression between different surrogate sugar treatments. Error bars represent the standard deviation (n=3). Different letters above the bars indicate significantly different values ($P < 0.05$).
